# Observations on the Structure and Functions of the Nervous System, Illustrated with Tables

**Published:** 1783

**Authors:** 


### LONDON MEDICAL JOURNAL,

For APRIL, MAY, and JUNE, 1783.

### SECTION I.

### BOOKS.

I. *Obſervations on the ſtructure and functions of the nervous ſyſtem, illuſtrated with tables. By* Alexander Monro, M. D. *Preſident of the Royal College of Phyſicians, and profeſſor of phyſic, anatomy and ſurgery in the Univerſity of Edinburgh. Folio,* Creech, *Edinb.* Johnſon, *London,* 1783. 104 pages, with 47 copper-plates, 2l. 12s. 6d. in boards.

SO long ago as the year 1779, our curioſity was greatly excited by an account publiſhed in the Edinburgh Medical Commentaries, of the diſcoveries of the learned profeſſor Monro, relative to the ſtructure of the nerves. In that account we were told, that the brain and nerves were found to be compofed not of ftraight fibres, as had till then been imagined, but of convoluted fibres, nearly $\frac{1}{9000}$ part of an inch in diameter, and which did not appear to be hollow, but folid. It was added alfo, that the profeffor, affifted by his microfcope, had difcovered throughout the whole vegetable and mineral kingdoms, a fyftem of convoluted fibres in every refpect analogous to the nerves of animals. But in the work now before us, the learned author very candidly acknowledges, that when he had extended his obfervations not only to the vegetable, but to the mineral kingdom, and more coolly and carefully confidered every circumftance, he began to fufpect fome optical deception. When he examined melted wax, he faw no fuch appearance; but at the very inftant of their becoming fomewhat opake by cooling, he faw the mafs fhoot into ferpentine fibres. In like manner all the metals when melted and allowed to cool feemed to be compofed of fuch fibres. But it having been fuggefted to him, by one of his friends, to compare gold, beaten into exceedingly thin leaves, with gold melted and cooled again, he found no difference in their appearance; whereas if the gold really confifted of ferpentine fibres, thefe by the heating, fhould have have become broader, and, of courfe, fhould have appeared larger and lefs numerous in the fame fpace. Again, he found that tallow, or any other foft fubftance, had the fame appearance, after being fpread, with a knife, upon a plate of glafs, as when, after being melted, it was allowed to cool upon the glafs. Yet although, after being melted and cooled upon the glafs, it had fhot into ferpentine fibres, in the fame manner as falts fhoot into chryftals, the figures of thefe fhould have been deftroyed, or altered, by the mechanical preffure of the knife.

Dr. Monro fufpects that this deception, produced by the microfcope, has mifled other anatomifts, particularly the late Mr. Hewfon and Mr. Falconer. The latter in his Experimental Enquiries, plate iv. fig. 4. has delineated cells, fuch as are faid to exift in the lymphatic glands, which cells, our author apprehends, will be found to exift only in the microfcope. As a decifive proof that a miftake was committed by thefe authors about the fpleen, he adds, that in fig. 2. of the fame plate, the veficles of the red particles of the blood, viewed through a lens of one twenty-third of an inch focus, are delineated nearly double the diameter of the cells of the fpleen, though viewed by a lens one fiftieth of an inch focus; fo that one veficle of the blood is reprefented by Mr. Falconer himfelf above fixty times the fize of one of the cells of the fpleen, within which, he teaches, it was contained and formed.

Of the tables, which compofe a principal part of the work before us, we fhall not attempt to give a defcription. That they are correct reprefentations of the appearances they are intended to delineate, we can have no doubt, from the knowledge we have of the great accuracy and fkill of the learned author in every thing that relates to anatomical fcience. We fhall therefore confine ourfelves to his obfervations, felecting, for the information of our readers, fuch as feem to be the moft interefting, and following the order of the chapters into which the work is divided.

*Circulation of the blood within the head*—It has been remarked by different writers, that the force of the blood fent to the head is broken by its afcent; by the angles at the rife of the internal carotid and vertebral arteries; by the turns which thefe make in their courfe; and by the uncommonly great proportion which the fum of the areæ of the branches bears to the area of the trunk.

trunk. But this intention of nature—Dr. Monro obſerves—appears more evidently in the ruminating quadrupeds; for he finds that in them a ſubſtance connected with the internal carotid artery, by Galen named *Rete mirabile* and by Heiſter *Plexus vaſorum et fibrarum uſus incogniti*, conſiſts entirely of a diviſion of that artery into ſmaller ſerpentine branches, which are afterwards collected, at the ſide of the ſella turcica into a trunk that is divided as in man. This plexus, in a fœtus calf, is the ſubject of the firſt plate.

This appearance in ruminant animals has not paſſed unnoticed by Haller, who was aware that it is formed ſolely by the ramifications of the carotid, as the reader will ſee by turning to his *Elem. Phyſ.* lib. x. ſect. 5. where he deſcribes it as being "verum arteriarum ramoſarum inter-"textarum plexum, in quas carotis reſol-"vatur."

Dr. Monro obſerves, that Keil and others by comparing the areæ of the internal carotid and vertebral arteries with the area of the trunk of the deſcending aorta, inſtead of comparing them with ſimilar branches ſent off from that trunk, have fallen into a miſtake with regard to the proportion of blood circulated in the head.

They

They have calculated it to be about one-fifth, but our author ſuppoſes it not to exceed one-tenth of the whole maſs. He preſents us with ſome judicious obſervations on the uſes of the ſinuſes of the brain, and relates an experiment to prove that in animals killed by hanging, death is not, as Petit has ſuppoſed, owing chiefly to the preſſure on the veſſels of the brain, but that it depends on the ſtoppage of reſpiration. After cutting a hole in the trachea of a dog, the animal was ſuſpended by a rope fixed round his neck above the hole, for three quarters of an hour, without depriving him of ſenſe or motion; but when he was afterwards ſuſpended with the rope below the hole, for a quarter of an hour, he became inſenſible and did not recover.

*Of the membranes of the brain*—Dr. Monro obſerves, that there are fewer veſſels diſperſed within the ventricles than upon the ſurface of the brain.

*Of the communication of the ventricles of the encephalon with each other*—The communication of the lateral ventricles with each other has never been clearly aſcertained, and has been doubted by ſome of the moſt reſpectable modern anatomiſts. Such a communication, however, is here pointed out by the learned profeſſor, and delineated neated in his 2d, 3d, and 4th tables. It is de-ſcribed as a hole large enough to admit a gooſe quill, ſituated under the fore part of the fornix. He obſerves, that the choroid plexuſes of the lateral ventricles are connected together by a broad vaſcular membrane, which adheres cloſely to the fornix above, and to the thalami nervorum opticorum below, covering and ſhutting the hole called anus, ſo as to prevent the lateral ventricles from communicating with each other, or with the third ventricle, at any place but that juſt now deſcribed.

Our author denies that the bottom of the fourth ventricle has any ſuch communication with the cavity of the ſpinal marrow, as Haller ſuppoſed, it being completely ſhut by its choroid plexus and pia mater. He has obſerved in the bodies of every one of fifteen children who died from internal hydrocephalus, that all the ventricles were diſtended; that on cutting into one of the lateral ventricles, all the ventricles were emptied, but that in none of them, water was contained in the cavity of the ſpinal marrow, or between its pia and dura mater.

*Of the abſorbent veſſels of the encephalon, and of the infundibulum, and glandula pituitaria*—Although lymphatics have never yet been demonſtrated

ſtrated in the brain of man or quadrupeds, the learned author thinks there is no juſt ground for doubting of their exiſtence. He is convinced by repeated experiments that the infundibulum is a hollow membranous tube, painted with many veſſels. He quotes Haller among the authors who have doubted of the hollowneſs of this part; but the doubts of that celebrated writer, on this ſubject, expreſſed in the firſt edition of his Phyſiology, printed in 1762, were removed before the publication of his laſt edition of the ſame work in 1778, for in the latter (lib. x. ſect. 1.) he ſays, "Cl. Profeſſor Upſalienſis J. Ad. Murray nuper ſummo ſtudio in hanc infundibuli "caveam inquiſivit; reperit pia membrana fieri "et arachnoidea, et interpoſita celluloſitate; "deſcendendo fieri graciliorem, inde iterum "lateſcere; cavum porro et glacie manifeſto re-"pleri, celluloſo textu carens. Dividi demum "in duos ramos, quorum quilibet ſuum glan-"dulæ pituitariæ lobum adeat. *Cum iſto adeo* "*viro, qui peculiari labore in hanc inquiſitionem* "*incubuerit, putaverim nos ſtare debere, ut infun-* "*dibulum nomen ſuum mereatur cavumque ſit.*" —In different tables Dr. Monro has delineated the infundibulum as it appears in man, the ſheep, and the ox.—He conjectures that the glanglandula pituitaria performs an office ſimilar to that of a conglobate gland.

*Of the uſe of the ventricles of the encephalon*—To the conjectures propoſed by authors, few of which are ſatisfactory, the learned Profeſſor only adds, that the ventricles ſerve to increaſe the ſurface of the pia mater.

*Of the cineritious and medullary ſubſtance of the brain*——The moſt ſuccefsful injection, we are told, is far from ſhewing, as Ruyſch and others have pretended, that the cineritious ſubſtance of the brain is entirely compoſed of veſſels. Dr. Monro, however, has not in any animal, obſerved in it regularly ſhaped bodies which might be ſuppoſed glandular. A great deal of cineritious matter is to be found incloſed within the medulla. In the middle ſubſtance of the brain and cerebellum, and even within the crura cerebri and cerebelli, or the tuber annulare & medulla oblongata, which are generally conſidered as pure medullary cords, he has found a great quantity of cineritious matter, into which after a good injection many veſſels were ſeen to penetrate. He conjectures, that the numerous veſſels which penetrate the medulla may ſerve, among other uſes, to ſupply the deep-ſeated cineritious matter, and alſo that in caſes of loſs

 of of ſubſtance of the cortex by wounds or otherwiſe, the veſſels which run from within, outwards, may not only furniſh matter to nouriſh the deep parts, but alſo to ſupply the loſs of the ſuperficial, in the ſame manner as when a bone has exfoliated, the inner parts live, and the outer are, perhaps, in part ſupplied by the veſſels which run from the canal of the marrow, outwards.

*Of the ſuppoſed origin or formation of the nerves*—Haller and others have ſuppoſed, that the whole medullary ſubſtance of the brain is employed in the formation of the nerves of the head, and of the ſpinal marrow. On theſe points Dr. Monro very ingeniouſly obſerves, that many medullary fibres of the brain appear, from their tranſverſe direction (delineated in his 5th and 7th tables) better calculated to connect the different ſides and different parts of the brain to each other, than to connect the brain to the nerves—that fiſhes and other animals with very ſmall brains, feel as accurately, and exerciſe their muſcles as violently as the other claſſes of animals in which the brain is proportionally much larger; that the brain of a man is, in proportion to his weight, 24 times heavier than that of an ox, and yet the nerves of the latter are, in their ſize, proportioned to the the bulk of his muſcles, and thoſe of the organs of his ſenſes, as of the eye and noſe, are proportioned to the extent of theſe organs, the olfactory nerve of an ox, for example, being many times larger than that of a man. Hence he is led to conſider the brain as a medium between the mind and the reſt of the body of the animal; by the intervention of the machinery of which the intellectual powers are influenced in a way we neither do, nor, probably, ever ſhall be able to comprehend; and that, in man eſpecially, a ſmall part only of it is lengthened out ſo as to give origin to the nerves. He even doubts whether inſtead of conſidering the brain as the origin of the nerves, we ought not to conſider it merely as connected with the nerves. He relates ſome facts, which ſeem to ſhew that the nerves may exiſt independent of the brain.

*Of the ſtructure of the ſpinal marrow*—We here meet with ſeveral curious anatomical remarks. An accurate deſcription and engraving are given of the ligamentum denticulatum. Dr. Monro finds that the right and left ſides of the ſpinal marrow are leſs intimately connected than what has been commonly imagined. This circumſtance, he thinks, explains the cauſe of hemiplegia.

*Of the pia mater, the colour and texture of the nerves*—The nerves have been generally conſidered as a continuation of the medullary ſubſtance of the brain and cerebellum, but the learned author finds, that, with a few exceptions, particularly of the optic nerves and portio mollis of the auditory, they are all of a browner colour than the medullary ſubſtance, their pia mater ſeeming to furniſh a quantity of cineritious matter. This circumſtance, we are told, is more obſervable in the nerves of the ſpinal marrow, than in thoſe of the head.

*Of the appearance of the nerves in their courſe, and particularly of their folds and joints*—Dr. Monro has found that in any of the four claſſes of large animals, the nerves, when viewed carefully with a common magnifying glaſs, appear to conſiſt of a ſemipellucid ſubſtance, in which a more white and opaque fibrous looking matter ſeems to be diſpoſed in tranſverſe and ſerpentine lines. This appearance is the ſubject of different figures in his 13th table. Theſe ſpiral fibres, which are beſt ſeen when the nerve is fully relaxed, are conſidered by our author as folds or joints in the nerve, ſerving to accommodate it to to the different ſtates of flexion and extenſion. As a proof that this is their chief uſe, he obſerves, that the tendons of all animals, in their relaxed ſtate, have a ſimilar appearance. Theſe joints, we are told, are nearly as numerous and as diſtinct in the nerves within the head as in the nerves of the muſcles. The ſpinal marrow likewiſe, it ſeems, has a number of tranſverſe furrows, which evidently ſerve the purpoſe of joints or folds.

*Of the plexuſes of nerves*—In the plexuſes, our author has found, that the fibres of the different trunks are intermixed, and every nerve under the plexus conſiſts of fibres of all the nerves, which were tied together above its origin from the plexus. This ſtructure is delineated in the 15th, 16th, and 17th tables.

*Of the connection of nerves which run in oppoſite directions, ſo as to be joined by their ſmall branches*—The moſt remarkable connection of this kind, we are told, is in the human face, where the portio dura of the auditory nerve is joined to the ſecond and third branches of the fifth pair of nerves. This is the ſubject of the 19th table.

*Of the connection of the ſeveral cords which compoſe each of the nerves*—The author has obſerved, that in the whole extent of the nervous ſyſtem, the the ſubordinate cords, of which the particular nerves conſiſt, form within their proper ſheaths a ſucceſſion of plexuſes, in which their fibrils are intermixed and combined again, in nearly the ſame manner as in the axillary plexus. This is delineated in the 18th table.

*Of the external covering of the trunks of the nerves, and of the cords of the funiculi of which they conſiſt*—Zinn, and after him Haller and others, have contended, that the dura mater does not accompany the nerves in their courſe, but that their external coat is merely a tough cellular ſubſtance. But Dr. Monro doubts whether this opinion is well founded. He obſerves, that the outer covering of the nerves in their courſe agrees with the dura mater in its toughneſs, colour, and fibrous texture, and probably in its properties, and that it cannot, as Zinn repreſents, be readily diſſolved into cellular threads. He obſerves, that in the greater number of nerves the funiculi or ſmaller cords have a ſimilar tough, denſe, fibrous coat, and within it a thin vaſcular pia mater.

*Of the ganglia of the nerves*—The ganglia of the ſpinal nerves are deſcribed by authors as being formed after the anterior and poſterior faſciculi of nervous fibres from the medulla ſpinalis are are united; fo that every nervous fibre from the fpinal marrow is fuppofed to pafs through a ganglion. But Dr. Monro has obferved, that the pofterior fafciculus only of the fpinal nerves enters into the ganglion; and that preceding anatomifts have been deceived by not having flit open the external coat of thefe nerves. One half, therefore, of all the nerves of the mufcular organs of the trunk of the body, and one half of the nerves of the arms and legs, do not pafs through ganglia.

Our author finds, that the nervous threads in ganglia are intermixed; and, having obferved that the yellowifh or brownifh matter of ganglia has numerous veffels conveying red blood difperfed upon it, and that its colour, efpecially in man, very much refembles that of the cortical fubftance of the brain, he is led to confider the ganglia as fources of nervous matter and energy. As to the greater hardnefs of the ganglia than of the cortical fubftance, which, to Dr. Meckel, has appeared to afford fufficient reafon for denying that they ferve for fecretion, this, our author apprehends, will evidently appear neceffary to defend them from external violence, mufcular preffure, &c. Upon the whole, he thinks it appears that all the nerves which iffue from ganganglia are formed by a combination of threads from many fources; and that the nerves in their paffage through a ganglion feem to receive new energy from the vafcular matter of the ganglion. This he fuppofes to be the reafon why ganglia are moft numerous in the nerves of organs of chief importance; as in thofe of the heart or of the inteftinal tube. The ftructure and appearance of ganglia in different animals is illuftrated by different figures in the 20th, 21ft, 22d, and 23d tables.

*Of fpheroidal bodies which, in fome animals, make a part of the nervous fyftem*—Thefe appearances, which are obfervable, it feems only in fifhes of the genus *Gadus* of Linnæus, viz. in the cod, whiting, and haddock, are delineated in the 32d, 33d, and 34th plates. The learned author does not venture to hazard any conjecture concerning their ufe. At firft he fuppofed they might fupply the place of ganglia, which he found wanting at the roots of their fpinal nerves: but in other fifhes, he obferves, there feems to be a fimilar defect of fuch ganglia.

*Of fome principal nerves which have not been properly traced by authors*—Zinn and Haller have defcribed the olfactory nerves, as being fo foft and fo fuddenly diffufed upon the membrane of the the nofe, that it is impoffible to trace them diftinctly by diffection. But our author affirms, that even in the human fubject he has been able to trace them a great way within the nofe. In the fheep and ox, it feems, thefe nerves are hollow.

Dr. Monro obferves, that the retina ends abruptly, like the edge of a tea-cup, fomewhat farther back in the eye than the ciliary circle, fo that it covers that part only of the bottom of the eye, on which the pictures of objects can be diftinctly painted.—He corrects an error in Meckel's treatife on the 5th pair of nerves, where the trunk of the internal carotid is reprefented as paffing between the fecond and third branches of that nerve, inftead of the inner fide of both.—He has been able to trace the nerves of the teeth, and obferves, that in a child at birth their feveral branches can be plainly fhown, firft connected fo as to form a plexus, and after that entering the pulp of the tooth. Other branches, he adds, pafs between the teeth to the gums.

On diffecting the nerves of the human larynx, our author has found that the recurrent and fuperior laryngeal nerves form a plexus, and that the mufcles of the larynx receive branches from each of thofe nerves. This explains why the voice is not entirely loſt by dividing the recurrent nerves.

Haller has denied that any nerve can be traced into the ligament of a joint, but Dr. Monro finds that the ligament of the wriſt receives a branch from the muſcular ſpiral nerve of the arm.—He is of opinion, that the chorda tympani is formed by the ſecond branch of the fifth pair, as well as by the portio dura of the ſeventh.—Laſtly, he has traced the progreſs and termination of the portio mollis of the ſeventh pair in the cochlea of the human ear.

*Of the appearance of the nerves viewed with a microſcope*—This chapter relates to the microſcopic deception mentioned at the beginning of this article. The appearances exhibited by different ſubſtances, animal, vegetable, and mineral, when viewed through a microſcope of great magnifying power, are delineated in ten different tables. Theſe plates were probably finiſhed before the author had found out his miſtake, otherwiſe, we preſume, a ſingle engraving would have been thought ſufficient for the purpoſe of illuſtrating this optical illuſion.

*Of the nature of the energy of the nerves*—This, and the remaining chapters, are almoſt entirely phyſiological. The learned profeſſor thinks we are are far from poſſeſſing poſitive arguments that the nerves operate by the medium of an electrical fluid. On the other hand, he obſerves, that the effects of compreſſion on the nerves of a ſound animal, and the experiment of producing repeated contractions of a muſcle by preſſing its nerve, after cutting it acroſs, ſeem to indicate that the energy depends on matter capable of being affected by ſimple preſſure.

*Whether the nerves convey the nouriſhment to our organs*—A variety of ingenious arguments are offered to prove, 1. That the arteries prepare and directly ſecrete the nouriſhment in all our organs. 2. That the nerves do not contain or conduct the nouriſhment, but by enabling the arteries to act properly, contribute indirectly to nutrition.

*Of ſenſation*—Dr. Monro conjectures that animals poſſeſs two kinds of feeling, one with and another without conſciouſneſs; the latter perhaps reſembling that kind of ſenſation which we muſt ſuppoſe inherent in vegetables.

*Of the termination of the nerves in the muſcular organs; and whether muſcles poſſeſs a vis inſita different from the vis nervea*—As proofs that muſcles or muſcular fibres ſeem to be organs

*sui generis*, not produced by the nerves, as some writers have supposed, but merely influenced by the energy they convey, the learned author observes, 1. That muscular fibres have considerable strength and toughness, whereas the nerves are pulpy and soft. 2. That the matter which we know to be medullary or nervous does not appear to be endowed with the power of contracting when irritated. 3. That as the nerves consist of threads laid parallel to each other, and which do not, like the blood vessels, divide into branches, it seems impossible to conceive that a small nerve can form a large mass of flesh; and lastly, that if the muscles were formed by the extremity of the nerves, they should shrink very remarkably on cutting the nerves, instead of which, he observed no sensible alteration in the muscles of the thigh and leg of a frog upwards of a year after he had cut across its spinal marrow or sciatic nerves.

As he has found from experiments, that the supposed vis insita of muscles is destroyed or excited by the same means as the vis nervea, he thinks it seems clearly to follow, that there is no just ground for supposing that any other principle, than the latter, produces the contraction of a muscle. If it should be objected that the the mufcles, when irritated, poffefs fome degree of vibration many days after their nerves have been cut, he thinks this may be explained by attending to the facts formerly mentioned, with the view of proving that the nerves, in their whole courfe, refemble the brain in ftructure, and as they proceed receive an addition of nervous energy.

*Of the manner and caufes of the actions of mufcles*—Many ingenious arguments are offered to prove that mufcular action cannot be accounted for on the yet known principles of mechanifm, and that the mufcular fibre varies its operation, according to the purpofe to be ferved. That, for inftance, when a mufcular fibre is punctured, it vibrates, which is the fitteft means of throwing off the offending caufe; that the alimentary canal, acted on gently by the food, performs a very complex periftaltic motion; that the abdominal mufcles act flowly and fteadily in expelling the contents of the rectum, but fuddenly and convulfively in vomiting; and that the bladder of urine, from which there is a fmall outlet, performs a flow and uniform contraction in difcharging its contents; whilft the heart contracts with a jerk.

The

The Stahlians have fuppofed that our mind, intimately acquainted with the texture of the body, reafons upon, and thereafter performs the feveral actions. On the other hand Dr. Whytt, who confiders the mind as the agent, denies that it underftands the texture of the body, and, therefore, makes a conclufion, not very intelligible, that the mind is *neceffitated* to act in certain ways. But our author obferves, that unlefs we fuppofe the mind to poffefs powers of which it has no confcioufnefs, thefe actions cannot be alledged to be directed by it; and farther, that unlefs we confider the mind as an intelligent being, poffeffing innate knowledge, or far more knowledge than it could have acquired by experience, we muft refer thefe operations to fome higher fource. The author therefore concludes with obferving, that when we reflect on the various effects of what has been commonly called the inftinct of animals, it feems to appear, "that the moft juft as well as the moft "becoming conclufion we can draw, is, that "the power which created all things, which "gave life to animals and motion to the hea-"venly bodies, continues to act upon, and to "maintain all, by the unceafing influence of a

"living

" living principle pervading the universe, the " nature of which our faculties are incapable of " duly comprehending."

